# Autoantibodies against insulin-like growth factor-binding protein-2 as a serological biomarker in the diagnosis of lung cancer

**DOI:** 10.3892/ijo.2012.1699

**Published:** 2012-11-15

**Authors:** YING ZHANG, XIA YING, SUXIA HAN, JING WANG, XIA ZHOU, E BAI, JIANYING ZHANG, QING ZHU

**Affiliations:** 1 Department of Oncology, The First Affiliated Hospital of Xi’an Jiao Tong University Medical Center, Xi’an, Shaanxi 710061, P.R. China;; 2 Department of Biological Sciences, The University of Texas at El Paso, El Paso, TX 79968, USA

**Keywords:** autoantibody, insulin-like growth factor-binding protein-2, biomarker, diagnosis, lung cancer

## Abstract

Insulin-like growth factor-binding protein-2 (IGFBP-2) is considered to be a human tumor antigen, and the tumor-specific immunity of IGFBP-2 has been reported in several types of cancer. The purpose of this study was to evaluate whether autoantibodies to IGFBP-2 can be used as diagnostic markers in lung cancer. The results demonstrated that serum anti-IGFBP-2 autoantibody levels were significantly elevated in lung cancer (mean, 1,633.318 ng/ml; median, 1,651.462 ng/ml; range, 342.732–4932.582 ng/ml) compared with benign lung disease (1,210.139, 1,035.900, 547.596–2,331.167 ng/ml) and normal controls (1,303.369, 1,194.800, 528.200–2140.500 ng/ml). The sensitivity and specificity of anti-IGFBP-2 autoantibodies in diagnosing lung cancer was 73.2 and 60.6%, respectively. When serum IGFBP-2 and anti-IGFBP-2 autoantibody were used together in the diagnosis of lung cancer, it can increase the discriminative power for lung cancer with a sensitivity of 85.7% and a specificity of 57.5%. In conclusion, this study demonstrates that circulating anti-IGFBP-2 autoantibodies can be used as a potential biomarker in diagnosing lung cancer.

## Introduction

Lung cancer has become the most common malignant tumor and the major cause of cancer deaths worldwide. The overall 5-years survival rate for lung cancer is only 15% (1). The largest gain in life expectancy in lung cancer patients has been among those with localized disease versus those with regional or distant metastasis. However, due to the asymptomatic nature of the early disease and the lack of effective screening methods, only 19% of lung cancers are localized at the time of diagnosis (2). Although there has been significant progress in diagnostic tools at clinics including CT (computed tomography) scans, bronchoscopy and sputum analysis, none of these turns out to be an effective tool in early diagnosis of lung cancer. How to establish a methodology to identify the high-risk individuals for lung cancer remains to be investigated.

Cancer has long been recognized as a multi-step process which involves not only genetic changes conferring growth advantage but also factors which disrupt regulation of growth and differentiation. It is possible that some of these factors could be identified and their functions evaluated with the aid of autoantibodies arising during tumorigenesis. The heterogeneity in the molecular pathogenesis of human cancers must be taken into account in both the design and interpretation of studies to identify makers which will be useful for early diagnosis of cancer. Many studies have demonstrated that the cancer cells can generate a unique set of antigenic change which can be recognized by the immune system of patients themselves. These antigens are called tumor-associated antigens (TAAs), whose abnormal regulation or excessive expressed are closely related to tumorigenesis (3). The appearance of circulating autoantibodies against these TAAs is one of the important ways which are manifested by cancer patient’s immune system (4,5). These anti-TAA autoantibodies can magnify the signal when the tumor antigen expression is very low even when it is not able to be detected, suggesting that these autoantibodies may be reporters identifying aberrant cellular mechanisms in tumorigenesis and also served as immunodiagnostic markers for cancer detection, especially because of the general absence of these antoantibodies in normal individuals and non-cancer conditions. For example, autoantibodies to TAAs such as p53 and p16 were detected in 10–20% of most type of cancer patients (6,7), while autoantibodies to p62 were 21% positive in patients with hepatocellular carcinoma (8). Due to the fact that these autoantibodies do not exist or appear with a very low titer in the serum samples of healthy people, therefore, the autoantibodies may become potential biomarkers in the diagnosis of certain type of cancer.

Insulin-like growth factor-binding protein-2 (IGFBP-2) is one member of the insulin-like growth factor family (IGFs), and recently reported to be a tumor-associated antigen (9). IGFBP-2 is overexpressed in a majority of malignant tumors, including glioma (10) and colorectal (11,12), prostate (13), ovarian (14) and breast cancers (15). Although overexpressed IGFBP-2 is associated with increased tumor stages and grades (12), relapse, metastasis (14) and prognosis (16) in advanced cancers, it is less satisfactory in diagnosing early cancers. A recently published paper (17) first demonstrated that serum anti-IGFBP-2 antibody was significantly elevated in gliomas and colorectal carcinoma, suggesting that the detection of circulating anti-IGFBP-2 antibody may be a potential approach in diagnosing early cancers. In the current study, we aimed to evaluate whether serum IGFBP-2 and anti-IGFBP-2 antibody can be used as biomarkers in detection of lung cancer.

## Materials and methods

### Patients and samples

Blood samples (n=294) were prospectively collected from consented individuals under an Institutional Ethics Committee-approved study from the First Hospital of Xi’an Jiaotong University for patients with lung cancer (n=190; 55 lung cancers stage I–II and 135 lung cancers stage III–IV), from 2011 to 2012. In addition, 9 patients also consented to a follow-up evaluation of anti-IGFBP-2 antibodies. Sera from six patients with lung cancer were collected after surgery and chemotherapy, and sera from three patients were also collected after each cycle of chemotherapy. As control, sera from patients with benign lung disease such as phthisis, pneumonia and hamartoma (n=33) were obtained from individuals from the same hospital who were having annual health examinations and had no evidence of malignancy (n=71, see Table I).

The blood was drawn before surgery, chemotherapy or radiotherapy, blood samples were separated by centrifugation at 2,500 rpm for 8 min and stored at −80°C until further analysis. Electrochemical luminescence kits (Roche, Mannheim, Germany) were used to measure serum levels of carcinoembryonic antigen (CEA), cytokeratin 19 fragments (CYFRA21-1) and neuron-specific enolase (NSE) in 138 patients (98 lung cancer, 17 lung benign disease and 23 controls, see Table II). Cutoff values for CEA, CYFRA21-1 and NSE were 3.4, 3.3 and 15.2 ng/ml, respectively, used by the First Hospital of Xi’an Jiaotong University. The same sera were also used for IGFBP-2 and anti-IGFBP-2 antibody measurement. Immunohistochemical analysis (IHC) of IGFBP-2 expression was performed on tissue specimens, collected during surgical operation.

### Indirect ELISA for serum IgG antibodies against IGFBP-2

Serum IgG antibody against IGFBP-2 was assessed by indirect enzyme-linked immunosorbent assay (ELISA) as previously described (17). Briefly, Immulon 4HBX microtiter plates (Dynex), were coated overnight (at least for 24 h) with 50 *μ*l of highly purified, human recombinant IGFBP-2 protein (R&D Systems Inc., Minneapolis, MN) diluted in 50 ml carbonate buffer (Sigma-Aldrich Corp., St Louis, MO) to a concentration of 1.0 *μ*g/ml or carbonate buffer alone in alternating columns. The last column of wells was incubated with 50 *μ*l per well of serially diluted, purified human IgG (25, 100, 250, 500, 1,000 and 2,000 ng/ml) (R&D Systems Inc.) to provide a standard curve.

Plates were blocked with 5% bovine serum albumin (BSA) in phosphate-buffered saline (PBS), 50 *μ*l per well, for 2 h at room temperature, washed four times with PBST (0.1% Tween-20 in PBS), and then incubated with diluted (1/5) serum in 1% BSA/PBS, 50 *μ*l/well, for 2 h at room temperature with gentle shaking. Plates were washed 4 times again and 50 *μ*l per well of goat anti-human IgG-HRP (Invitrogen Ltd, Paisley, UK) diluted 1:30,000 in 1% BSA was added and incubated for 1 h at room temperature. Following four washes, 100 *μ*l per well of tetramethylbenzidine (TMB) was added, reaction was stopped by adding 100 *μ*l 2M sulphuric acid (H_2_SO_4_). Plates were then read at 450 nm. The optical density (OD) of each serum dilution was calculated as the OD of the protein-coated wells minus the OD of the buffer-coated wells. The concentration of serum anti-IGFBP-2 antibody was calculated based on the standard curve on each plate.

### Direct ELISA for serum IGFBP-2

Serum IGFBP-2 was measured with commercially available IGFBP-2 ELISA kits (RapidBio Laboratory, Calabasas, CA) following the manufacturer’s instructions. The concentrations of the IGFBP-2 standards for building a standard curve were 62.5, 125, 250, 500, 1,000 and 2,000 ng/ml. Serum was serially diluted.

### IHC analysis

IGFBP-2 immunostaining was carried out as described (10). The sections of tumor tissues were immunostained with monoclonal anti-IGFBP-2 antibody (Cell Signaling Technology, Boston, MA). Five most heavily stained fields were chosen to determine the percentage of positive cells. Zero represents no positive, + represents <10%, ++ represents 11%–30%, +++ represents 31%–60%, and ++++ represents >60% positive staining cells.

### Statistical analysis

Statistical analyses were carried out using SPSS 13.0. Demographic data were analyzed with χ^2^ test. The differences of anti-IGFBP-2 antibody and serum IGFBP-2 levels between controls and patients were assessed using a standard non-parametric Mann-Whitney U test. Receiver operating characteristic (ROC) curves were constructed to determine the discriminatory capacity of anti-IGFBP-2 antibodies for diagnosis, and the area under the curve (AUC) was analyzed by χ^2^ test. Sensitivity was defined as the number of patients with elevated anti-IGFBP-2 antibodies in serum divided by the total number of patients with lung cancer. Specificity was defined as the number of patients without elevated anti-IGFBP-2 antibodies in serum divided by the total number of control patients. Correlations of serum anti-IGFBP-2 antibody levels with tumor tissue IGFBP-2 expression levels were assessed by Pearson’s correlation analysis. The results were considered to indicate a statistically significant difference at p-value <0.05.

## Results

### Elevated serum anti-IGFBP-2 antibody levels in lung cancer

**Figure 1.**
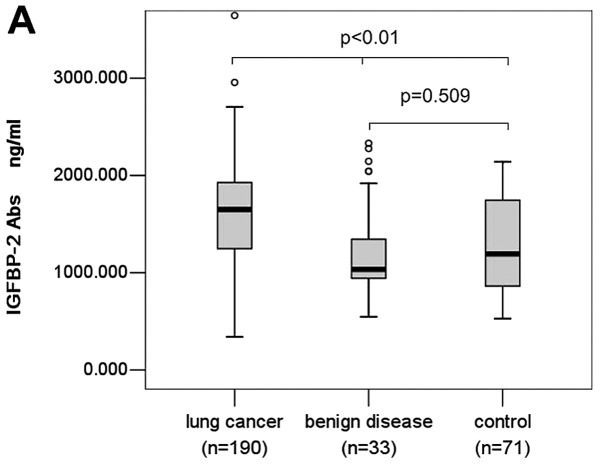

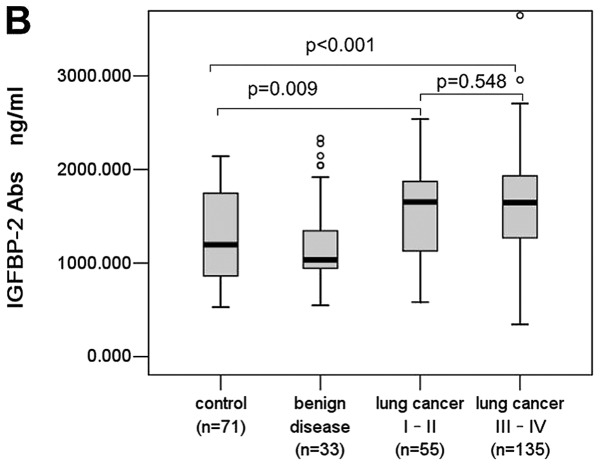
Serum IgG autoantibodies to IGFBP-2. (A and B) Comparisons among normal controls, benign lung disease and patients with lung cancer, and sub-stages. Median levels of serum autoantibodies to IGFBP-2 and interquartile ranges are illustrated by box plot. N, number of subjects; dot, outliers; IGFBP-2, insulin-like growth factor-binding protein-2.

Serum levels of anti-IGFBP-2 antibodies were determined by ELISA as described in the section of Materials and methods, the study population is summarized in Table I. We found serum anti-IGFBP-2 antibody level of lung cancer patients (mean, 1633.318 ng/ml; median, 1651.462 ng/ml; range, 342.732–4932.582 ng/ml) was significantly higher than that of patients with benign lung disease (mean, 1210.139 ng/ml; median, 1035.900 ng/ml; range, 547.596–2331.167 ng/ml) and normal controls (mean, 1303.369 ng/ml; median, 1194.800 ng/ml; range, 528.200–2140.500 ng/ml) (P<0.001, Fig. 1A). However, there was no significant difference between serum anti-IGFBP-2 antibody levels of patients with lung benign disease and normal controls (P= 0.509, Fig. 1A).

Li *et al*(17) demonstrated that anti-IGFBP-2 antibody levels were higher in early cancers than advanced cancers in gliomas and colorectal cancer. When stratifying the analysis by American Joint Committee on Cancer stage for lung cancer, interestingly, a modest difference in anti-IGFBP-2 antibody levels was observed between early cancers and advanced cancers in lung cancer. Patients with lung cancer stage I–II (mean, 1549.671 ng/ml; median, 1651.749 ng/ml) did not have an elevated anti-IGFBP-2 antibodies compared to patients with lung cancer stage III–IV (1667.397 ng/ml; median, 1648.400 ng/ml) (P= 0.548, Fig. 1B). There was no significant association of the serum levels of anti-IGFBP-2 antibodies in any of the collected histological types (P= 0.701) (data not shown).

### Serum anti-IGFBP-2 antibodies as biomarkers for lung cancer diagnosis

**Figure 2.**
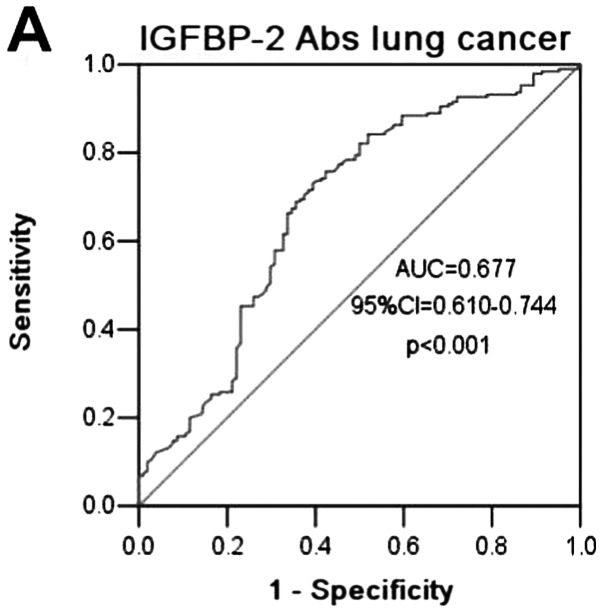

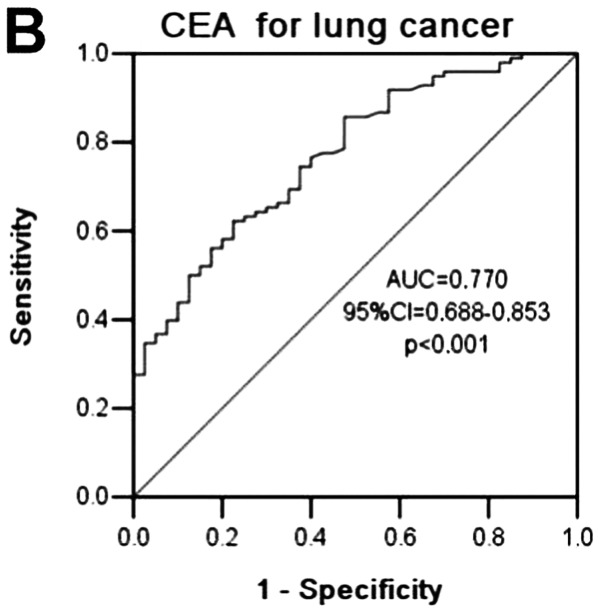

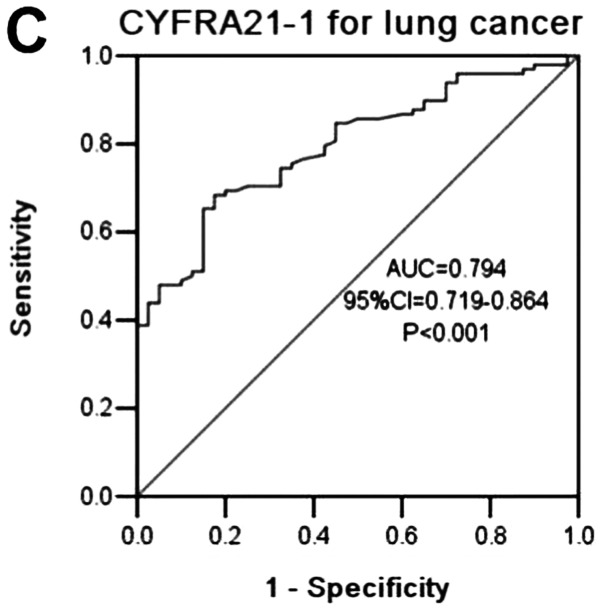

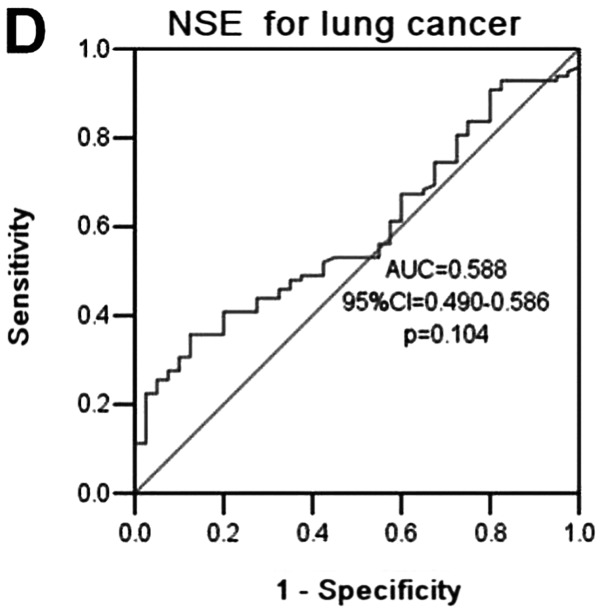

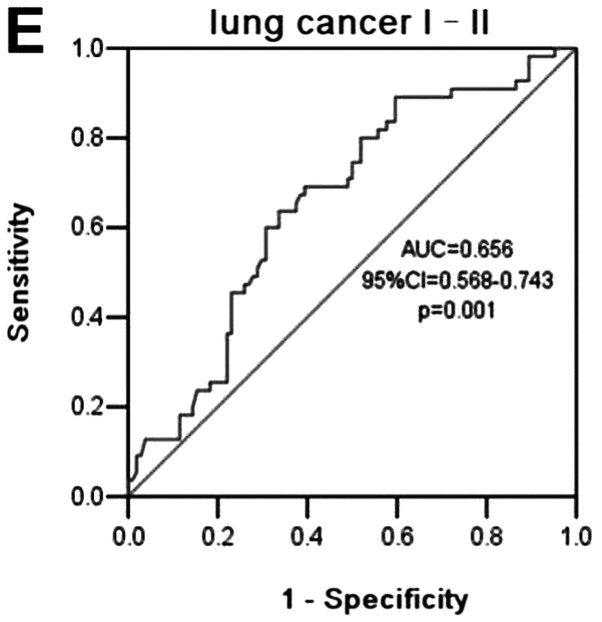

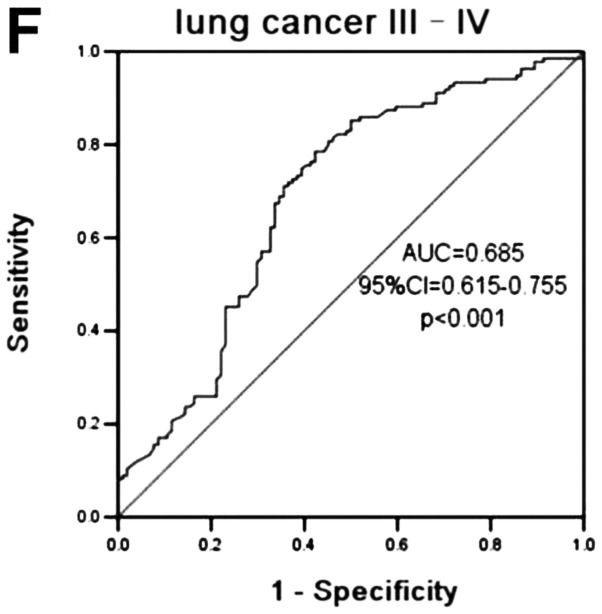
ROC curves of the serum anti-IGFBP-2 antibodies, CEA, CYFRA21-1 and NSE in discriminating between controls and patients with lung cancer. (A–D) ROC curves of the serum anti-IGFBP-2 antibodies, CEA, CYFRA21-1 and NSE; (E) ROC curves of the serum anti-IGFBP-2 antibodies in discriminating lung cancer I–II; (F) ROC curves of the serum anti-IGFBP-2 antibodies in discriminating lung cancer III–IV. CI, confidence interval.

ROC curves were constructed between lung cancer and benign lung disease and normal controls. The AUC of using anti-IGFBP-2 antibodies as diagnostic biomarkers for lung cancer was 0.677 (95% CI=0.610–0.744; P<0.0001) (Fig. 2A), in contrast to an AUC for CEA, CYFRA21-1 and NSE of 0.770 (95% CI= 0.688–0.853, P<0.0001), 0.794 (95% CI= 0.719–0.864, P<0.0001) and 0.588 (95% CI= 0.490–0.586, P= 0.104), respectively, for cancer in our patient cohort (Fig. 2A–D). When stratifying lung cancer by tumor stage, the AUC for lung cancer I–II and lung cancer III–IV was 0.656 (95% CI=0.568–0.743; P= 0.001) and 0.685 (95% CI= 0.615–0.755; P<0.001) (Fig. 2E and F), respectively, indicating some ability of anti-IGFBP-2 antibodies to diagnose lung cancer. Sensitivity, specificity, and all cutoff values of anti-IGFBP-2 antibody levels were determined using ROC analysis. The anti-IGFBP-2 antibody cutoff values of 1,151.351, 1,261.208, 1,448.053, 1,651.606, 1,922.826, 2,029.312 ng/ml yielded sensitivities of 80, 73.7, 61.6, 49.5, 25.3 and 16.3%; and specificities of 50, 59.6, 67.3, 71.2, 83.7 and 89.5%, respectively. Based on these data, a level of 1,264.306 ng/ml (the sum of sensitivity and specificity was the highest) was determined to be the most efficient threshold and we set this level as the cutoff value. The sensitivity of the assay was 73.2% and the specificity 60.6%, and the positive and negative predictive values were 77.2 and 55.3%, respectively. The sensitivity of serum anti-IGFBP-2 antibodies in the detection of lung cancer was higher (73.2%) than that of CEA (45.9%), CYFRA21-1 (65.3%), or NSE (50%), (P<0.001, P=0.166 and P<0.001, respectively) although the differences were not significant (P<0.001, P=0.166 and P<0.001, respectively).

### Combination of serum IGFBP-2 and anti-IGFBP-2 antibodies can increase the efficacy of cancer diagnosis

**Figure 3.**
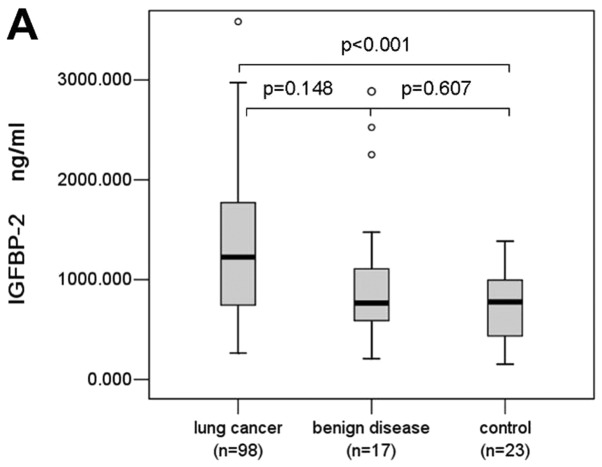

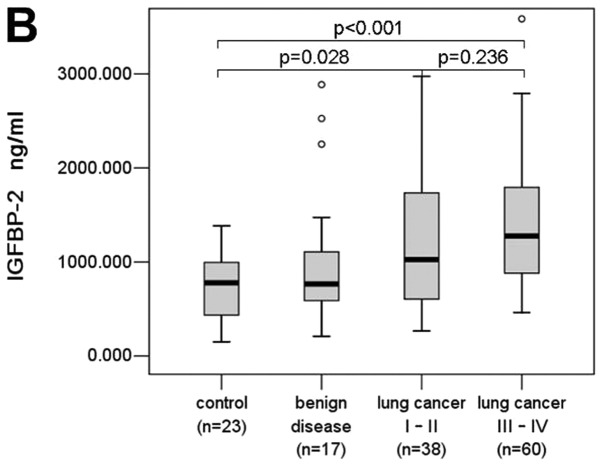

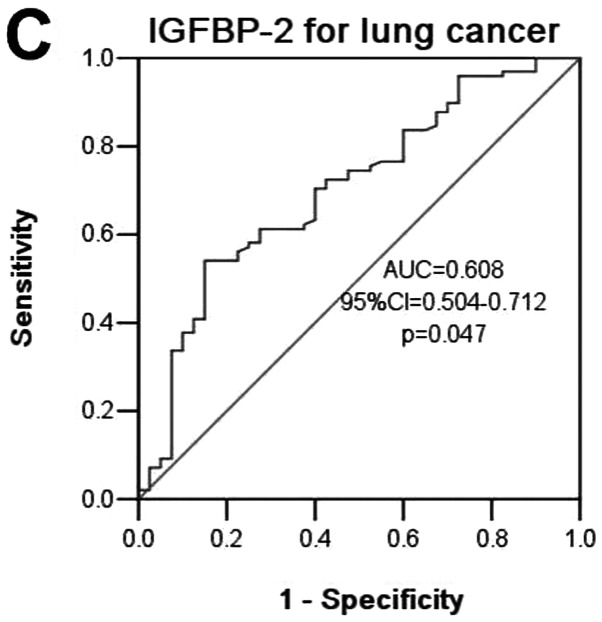
Diagnostic values of combined serum IGFBP-2 and anti-IGFBP-2 antibodies in distinguishing between controls and patients with lung cancer. (A and B) Serum IGFBP-2, comparisons among normal controls, benign lung disease and patients with lung cancer, and substages. (C) ROC curves of using serum IGFBP-2 in discriminating lung cancer. Median levels of serum autoantibodies to IGFBP-2 and interquartile ranges are illustrated by box plot.

In this study, we also detected serum IGFBP-2 levels in 98 patients with lung cancer, 17 patients with benign lung disease, and 23 normal controls, and found that serum IGFBP-2 levels were significantly elevated in lung cancer patients (mean, 1,304.273 ng/ml; median, 1,225.109 ng/ml; range, 265–3,584.674 ng/ml) compared with controls (749.428, 777.228, 51.944–1,384.217 ng/ml) (P<0.0001, Fig. 3A). Serum IGFBP-2 levels in patients with lung cancer were higher than that of benign lung disease (1,036.015, 766.304, 209.185–2,885.543 ng/ml), but the difference was not significant (P=0.148, Fig. 3A). There was no significant difference between lung cancer I–II and lung cancer III–IV either (P= 0.236, Fig. 3B). We conducted ROC curve analyses using serum IGFBP-2 levels in discriminating between patients and controls (Fig. 3C). The AUC of using IGFBP-2 as diagnostic biomarker for lung cancer was 0.608 (95% CI=0.504–0.712, p=0.047), the sensitivity and specificity were 54.1 and 72.5%, respectively, with the cutoff value of 1,173.033 ng/ml. We found that the combination of serum IGFBP-2 and anti-IGFBP-2 antibodies can augment the discriminative power for lung cancer with the sensitivity of 85.7% and the specificity of 57.5%.

### Relationship of serum anti-IGFBP-2 antibodies and tumor IGFBP-2 expression in lung cancer

**Figure 4.**
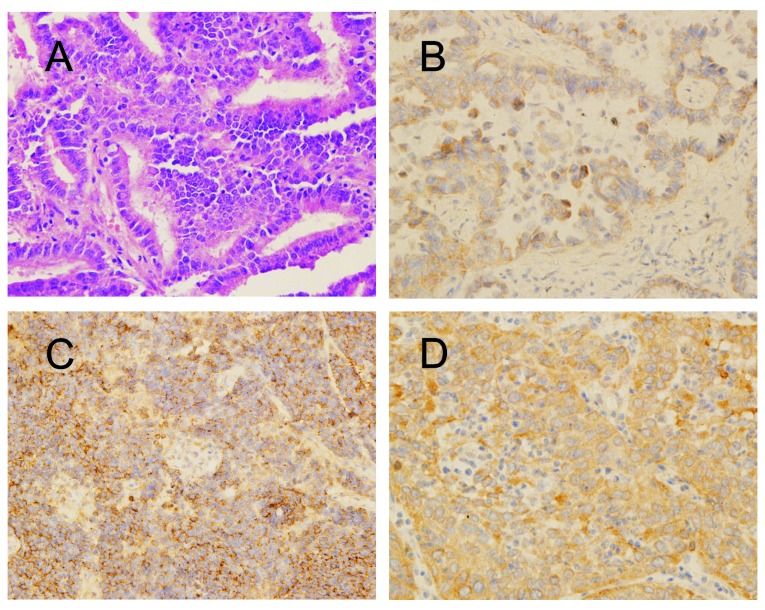
IGFBP-2 expression in lung cancer tissue sections. (A) Lung cancer tissue, Hematoxylin and eosin; (B) IGFBP-2 expression in lung adenocarcinoma; (C) IGFBP-2 expression in SCLC; (D) IGFBP-2 expression in lung squamous cell carcinoma; magnification, ×400.

In this study, we also examined IGFBP-2 expression in 23 lung cancer tissue sections (Fig. 4), and found 69.6% (16/23) of the lung cancer specimens had low (43.5%) to high (26.1%) IGFBP-2 expression, and 82.6% (19/23) of the tissue specimens had anti-IGFBP-2 antibody levels above the cut-off value of 1,264.306 ng/ml (data were not shown). Interestingly, we did not find a correlation between serum anti-IGFBP-2 antibody level and tumor IGFBP-2 expression level in lung cancer.

### Follow-up of serum levels of anti-IGFBP-2 antibodies in lung cancer

In order to determine whether concentration of anti-IGFBP-2 antibodies would change with the clinical treatment, a follow-up study has been performed. In the current study, 6 patients were observed, and serum samples were collected from these patients at 3–4 weeks after tumor resection, and for some patients samples were also collected after 1–2 cycles of postoperative chemotherapy. Data from three patients with chemotherapy instead of surgical resection were also analyzed (Table III).

**Figure 5.**
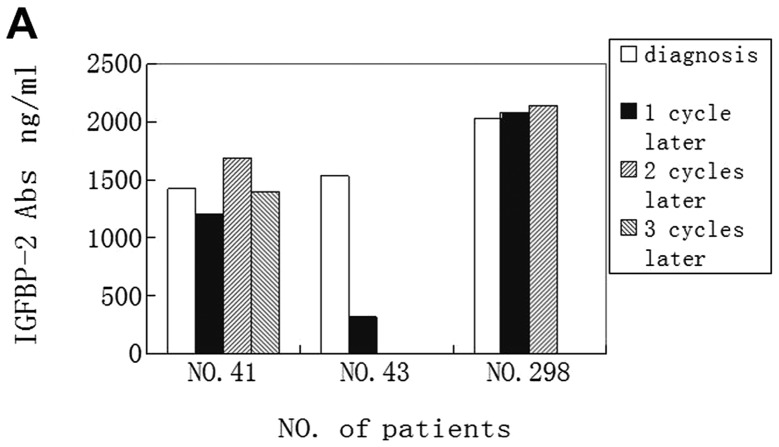

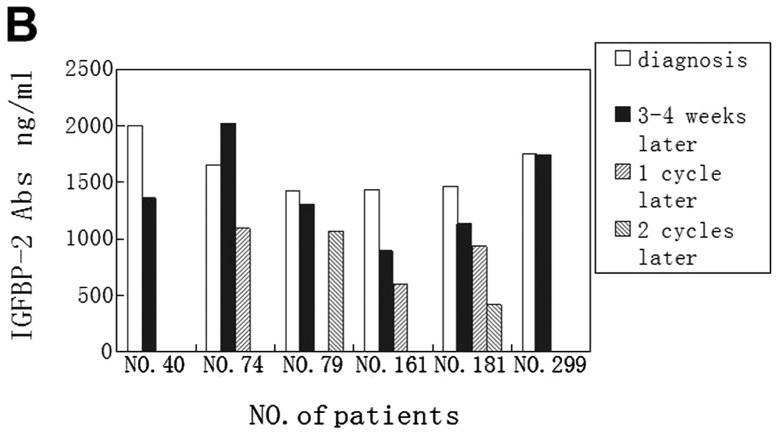
(A) Three patients with unresectable disease who underwent chemotherapy during follow-up; PD, progressive disease. Patient no. 41 developed liver metastasis after 2 cycles chemotherapy of vinorelbine and cisplatin, and as patient no. 298, the tumor size increased after 1 cycle of docetaxel and cisplatin chemotherapy. (B) Levels of anti-IGFBP-2 antibodies in 6 sera from patients with lung cancer before and after radical procedure and during follow-up. The sample of 1 cycle postoperative chemotherapy of patients no. 79 was not collected, and for patient nos. 40 and 299, there were only samples of before and after radical procedure.

Anti-IGFBP-2 antibody level before operation was elevated in all of the 6 patients (100%). Interestingly, antibody level was decreased in 4 patients (66.7%) after 3–4 week treatment, and only one patient (16.7%) has an elevated antibody level, and one patient’s antibody level was not changed. We also found that anti-IGFBP-2 antibody levels in all patients were reduced with the time progression (Fig. 5B).

In the follow-up observation of three patients who underwent chemotherapy, levels of anti-IGFBP-2 antibodies were elevated in all of the 3 patients (100%) at diagnosis, levels decreased in 2 patients (66.7%) after 1 cycle chemotherapy, and slightly elevated in one patient whose disease progressed at that time. For patient no. 41, who developed a liver metastasis after 2 cycles of chemotherapy of vinorelbine and cisplatin, the antibody levels were also decreased when the disease progressed (Fig. 5A).

## Discussion

Previous studies have indicated that IGFBPs are modulators of IGF signaling through sequestration of IGFs. Soluble and membrane-associated IGFBP-2 directly binds to proteoglycans and integrins (18,19), suggesting that IGFBP-2 may be a negative or positive regulator of cell adhesion, migration, and invasion in an IGF-independent manner (20–24). In the same way, IGFBP-2 positively or negatively regulates cell growth and survival in certain types of cancers in both of *in vitro* and *in vivo* studies (20–22,25,26). Thus, increased IGFBP-2 confers advantage or disadvantage for tumor growth, depending on cell type and physiological conditions.

Despite these two opposite effects of IGFBP-2 on the biological behavior of cancers, studies on biochemistry and molecular pathology have demonstrated that IGFBP-2 is over-expressed in a wide variety of human malignancies, including lung cancer, glioma, prostate cancer, colorectal cancer, ovarian cancer, adrenocortical tumor, breast cancer and leukemia. Importantly, IGFBP-2 is frequently overexpressed in advanced cancers, suggesting that it may be involved in the metastatic process. Serum IGFBP-2 can be used for prediction of chemotherapy response and prognosis in ovarian cancer and acute lymphoblastic leukemia (27). Some other studies have also demonstrated that IGFBP-2 can be considered as a regulator of phosphatidylinositol 3-kinase (PI3K)/Akt/PTEN to promote tumor progression (19,21,28) and also as a p53 target (29). Grimberg *et al*(29) have reported that loss of IGFBP-2 can inhibit the ability of p53 to further activate extracellular signal-regulated kinase (ERK)1 by IGF-I. Migita *et al*(30) also found that intracellular IGFBP-2 regulates caspase-3 expression and contributes to the inhibitory effect on apoptosis independent of IGF in lung adenocarcinoma. Therefore, IGFBP-2 may offer a novel therapeutic target and serve as an anti-apoptotic biomarker for lung adenocarcinoma.

As mentioned above, the autoantibodies against TAAs can be used as reporters in identifying aberrant cellular mechanisms in tumorigenesis and also served as immunodiagnostic markers for cancer detection. Our results provide the first evidence that the serum levels of anti-IGFBP-2 antibodies in patients with lung cancer are higher than that of patients with benign lung diseases and normal controls. The majority of patients with lung cancer present with advanced disease because there are no symptoms at the early stage. Although CEA, CYFRA21-1 and NSE are commonly used markers in lung cancer diagnosis, none of these markers is optimal. The finding in this study may provide a potential marker of anti-IGFBP-2 antibody in diagnosing lung cancer. The sensitivity of the assay was 73.2% and the specificity 60.6% with the cutoff value of 1,264.306 ng/ml, which is better than CEA, CYFRA21-1 and NSE in lung cancer detection.

A recently published paper demonstrated that serum anti-IGFBP-2 antibody levels were significantly elevated in early cancer compared to advanced cancers in gliomas and colorectal carcinoma (17). Interestingly, our study suggested different results in lung cancer, indicating a different immunogenicity of IGFBP-2 in patients with lung cancer compared to patients with gliomas and colorectal carcinoma. It may be related to the microenvironment of the various tumors (31) and immunosuppressive mechanisms induced by tumor cells (32) and also a different role of TAAs in tumor development.

Due to the high specificity of the autoantibodies to TAAs in cancer, anti-TAA antibodies have been generally considered as reliable biomarkers in cancer. At the cut-offs of 1,264.306 and 2,029.312 ng/ml of anti-IGFBP-2 antibodies, the specificity for lung cancer were 60.6 and 89.5%, respectively. It is well known that if only one anti-TAA antibody is used as tumor marker, the sensitivity is about 10–20%. As described above, IGFBP-2 is frequently overexpressed in advanced cancers and can be used for prediction of chemotherapy response and prognosis in some malignancy. When serum IGFBP-2 and anti-IGFBP-2 antibodies were detected simultaneously, the sensitivity of the assay was raised to 85.7%, and the specificity was 57.5% indicating that use of both serum IGFBP2 and anti-IGFBP-2 antibody can increase the diagnostic efficacy in lung cancer.

Our study also found that most of patient serum levels of anti-IGFBP-2 antibodies (66.7%) were decreased after surgical operation and chemotherapy. When the tumor size was increasing or the patient was developing metastasis, the serum levels of anti-IGFBP-2 antibodies were increased, suggesting a role for anti-IGFBP-2 antibodies in assessing response to therapy in lung cancer.

The weakness of the study is that the healthy controls did not receive a bronchoscopy, possibly leading to misclassification of the study subjects, and there was also the relatively small sample size, especially for patients with benign lung diseases. Further studies with larger sample size from different type of cancer will be performed to confirm and validate whether anti-IGFBP-2 antibodies can be also used as a diagnostic marker in other type of cancer.

## Figures and Tables

**Table I.** Characteristics of patients.

	n (male/female)	Range of the age (years)	Mean of the age(years)
Normal controls	71 (49/22)	36–77	58.75
Benign lung disease	33 (20/13)	34–88	59.94
Lung cancers	190 (138/52)	27–82	61.38
Lung cancer I	21 (15/6)	42–82	65.67
Lung cancer II	34 (24/10)	38–76	59.88
Lung cancer III	73 (53/20)	27–82	60.52
Lung cancer IV	62 (46/16)	32–78	61.61
Total	294 (207/87)	27–88	60.55

Stratifying the analysis by American Joint Committee on Cancer stage for lung cancer.

**Table II.** Characteristics of patients.

	n (male/female)	Range of the age (years)	Mean of the age(years)
Normal controls	23 (16/7)	36–77	58.13
Benign lung disease	17 (13/4)	34–83	60.94
Lung cancers	98 (68/30)	35–82	59.63
Lung cancer I	17 (12/5)	42–82	66.06
Lung cancer II	21 (14/7)	38–76	58.19
Lung cancer III	40 (24/16)	35–79	58.35
Lung cancer IV	20 (18/2)	37–75	58.25
Total	138 (97/41)	35–83	59.54

Stratifying the analysis by American Joint Committee on Cancer stage for lung cancer.

**Table III.** Characteristics of patients for follow-up.

Patients	Age/sex	Stage	Pathology	Therapy
40	62/M	II B	Squamous	Radical procedure
74	45/F	II A	Adeno	Radical procedure
79	55/M	III A	Adeno	Radical procedure
161	41/F	II A	Adeno	Radical procedure
181	62/M	III A	Adeno	Radical procedure
299	48/M	III B	Adeno	Radical procedure
41	56/M	III A	Squamous	Chemotherapy (NP)
43	62/F	III B	Adeno	Chemotherapy (TP)
298	41/M	IV	Adeno	Chemotherapy (TP)

Squamous, squamous cell carcinoma; adeno, adenocarcinoma; NP, vinorelbine and cisplatin (vinorelbine for 25 mg/m^2^ and cisplatin for 75 mg/m^2^); TP, docetaxel and cisplatin (docetaxel for 75 mg/m^2^ and cisplatin for 75 mg/m^2^).

## Abbreviations:

IGFBP-2: insulin-like growth factor-binding protein-2
CT: computed tomography
TAAs: tumor-associated antigens
IGFs: insulin-like growth factor family
CEA: carcinoembryonic antigen
CYFRA21-1: cytokeratin 19 fragments
NSE: neuron-specific enolase
IHC: immunohistochemical analysis
ELISA: enzymelinked immunosorbent assay
IgG: intravenous gamma globulin
BSA: bovine serum albumin
PBS: phosphate-buffered saline
HRP: horse radish peroxidase
TMB: tetramethylbenzidine
H2SO4
OD: optical density
ROC: receiver operating characteristic
AUC: area under the curve
PI3K: phosphatidylinositol 3-kinase
Akt: serine-threonine kinase
PTEN: phosphatase and tensin homolog deleted on chromosome ten
ERK: extracellular signal-regulated kinase
